# Attitudes to Cannabis Use and Public Prevention Information Among Young Adults: A Qualitative Interview Study With Implications for Prevention Practice

**DOI:** 10.3389/fpubh.2022.830201

**Published:** 2022-06-23

**Authors:** Pia Kvillemo, Anna K. Strandberg, Johanna Gripenberg

**Affiliations:** Stockholm Prevents Alcohol and Drug Problems (STAD), Centre for Psychiatry Research, Department of Clinical Neuroscience, Karolinska Institutet, Stockholm Health Care Services, Stockholm, Sweden

**Keywords:** cannabis, youth, prevention, attitudes, intervention, law, policy, public information

## Abstract

**Background:**

Cannabis use carries an increased risk of ill health and social problems, especially when initiated at a young age. Drug use is influenced by individual beliefs, knowledge, and attitudes, which are, in turn, governed by social and environmental factors. In recent years, a less restrictive attitude toward cannabis has been observed in many countries, with concerns about increased cannabis use among young people. The aim of the current study was to gain a deeper understanding of young adults' attitudes toward cannabis use and public prevention information about cannabis.

**Methods:**

A qualitative interview study was conducted among 32 anonymous informants aged 18–29 years in the Stockholm region. Participants were recruited through purposeful sampling, and semi-structured in-depth interviews were conducted using a digital video calling platform. A qualitative content analysis of the interviews was performed to generate categories and codes for cannabis use and attitudes toward prevention information.

**Results:**

Both cannabis users and abstainers perceived some risks with cannabis; however, for many users, the positive effects appeared to outweigh any expected harm. Furthermore, the existing public information was perceived as less credible because of an excessive focus on harm. The informants expressed a desire for neutral facts about the effects of cannabis, delivered by credible senders. Moreover, they felt that prevention information should be delivered by individuals whom young people look up to or with whom they can identify, for example, people with authority or famous people such as influencers. The informants also underlined the importance of dialogue with the target group and taking young people's experiences into account when providing information about cannabis.

**Conclusion:**

Current risk awareness associated with cannabis use among young adults is insufficient to prevent them from using cannabis. Public prevention information should preferably combine a fact-based focus on risks with recognition of cannabis' short-term desired effects, delivered by credible senders with authority or those with whom young people can identify.

## Introduction

Cannabis use has been shown to increase the risk of health issues and social problems, especially in younger age groups (1–7). The general public health effects of cannabis use are still under debate, however there seem to be consensus on cannabis' association to several health problems and also that young people are vulnerable to cannabis due to a developing brain (8). Research has found that there is a link between cannabis use and schizophrenia, and that cannabis use increases the risk of premature death (9). Cannabis use has further been associated with chronic bronchitis and vascular conditions that increases the risk of cardiovascular diseases (10). In addition, cannabis use is related to negative psychological and cognitive consequences, especially among those who begin using it in adolescence (11). Cannabis negatively affects memory, language, and logical analytical ability (11, 12). Moreover, there is a risk of developing addiction, although this risk may vary depending on routes of administration, doses, forms of cannabis and setting (13). Estimations indicate that 10–20 percent of those using cannabis on a daily basis develop addiction (14) and that a ~10% of those who use cannabis even once develop an addiction (1). Prior research has repeatedly shown that low socioeconomic status is a risk factor for substance use and related problems (15, 16). However, recent research from Canada (17), the United States (18–20), Serbia (21), Switzerland (22), and Sweden (23) suggest that high socioeconomic status too is associated with excessive substance use among young people, although for other reasons, e.g., excessive pressures to achieve and isolation from parents (24).

Most countries worldwide prohibit the production, use, and distribution of cannabis for recreational use. In recent years, however, a less restrictive attitude has been observed, manifested in decriminalization, and even in legalization of production and sales (25). Various states in the USA, e.g., Alaska, Colorado, Oregon and Washington have legalized cannabis for personal use, and in Uruguay and Canada, retail and production systems were introduced in 2014 and 2018, respectively (26). In Europe, the policy on cannabis vary between countries and several of them have moved toward decriminalization of cannabis use or personal possession, e.g., Luxembourg, Croatia, Portugal and Slovenia, while the use of cannabis is still a punishable offense in Cyprus, France, Finland, Greece, Hungary, Norway and Sweden (27). In Sweden, both use and possession of cannabis is a criminal offense under the Narcotics Penal Code (28). In parallel with global liberalization of cannabis policy, people, including adolescents and young adults, report a less negative attitude toward cannabis, lowered risk awareness, and increased use (25, 29–35). When asked about reasons for using cannabis, users report a number of perceived positive effects, such as relaxation, anxiety management (35, 36), increased creativity or productivity, facilitation of social contact (35), relief of pain, and other symptoms, especially when using cannabis for medical purposes (37, 38), and less side effects than for example when using alcohol (38, 39).

Compared to other European countries, cannabis use in the general Swedish population is still fairly low (32, 40). Estimated prevalence the last 12 months among people 15–34 years of age in Sweden 7.5%. Corresponding figures for France are 21.8%, Spain 19.1% and Germany 16.9%. However, a recent population survey in Sweden showed that 17% of women and 25% of men aged 16–29 have tried cannabis at least once (34), indicating that cannabis use among adolescents and young adults remains a public health concern. The figures also show that cannabis use appears to be slightly more common among young men than among young women. International research shows that the attitudes to cannabis policy, or laws on regulation, differ between users and non-users, which from a public information perspective is important to bear in mind. In the 1990's, Skretting showed that only 65% of cannabis users in Norway were in favor of prohibition of cannabis compared to 95% of non-users (41). Some years later, a study in Huston in the USA showed that 68% of drug users were in favor of legalizing cannabis, while only 33% of the non-users expressed approval (42) and in 2008, a Dutch study showed that 7% of cannabis users were found to be in favor of cannabis prohibition, compared to 50% of non-users (43).

According to the social learning theory, proposed by Albert Bandura (44), both environmental and cognitive factors interact to influence human learning and behavior, emphasizing the importance of observing, modeling, and imitating the behaviors, attitudes, and emotional reactions of others (44–50). These factors are, in turn, influenced by the social environment, including family, friends, the local community, and culture, as well as laws and associated penalties. Further, the rational choice theory states that people's behavior is based on perceptions of the expected utility of a given choice in relation to possible detriments (51, 52). The perception of risks has been shown to play an important role in cannabis use (53–55). However, knowledge about potential harm alone has been insufficient in preventing cannabis use (55, 56). In a recent survey of 1,161 active athletes, Zieger et al. (56) found that attitudes mediated the relationship between knowledge and cannabis use. In another European study of 86,107 students aged 15–16 years, Piontek et al. (49) similarly highlighted the importance of personal attitudes for cannabis use, based on the finding that the immediate social situation, for example, friends' behavior, was associated with cannabis use. The importance of friends' behavior for substance use was also supported in a recent qualitative interview study among affluent Swedish students aged 15–19, which showed that social availability of the substance and a desire to fit in at parties was a central motive for substance use (57).

Although a lot of research on mental health promotion interventions, including interventions related to substance use, has been carried out (58), technical advances and new generations of young people continuously growing up, make updated knowledge on the best way to effectively raise risk awareness and reduce positive attitudes toward cannabis among young people necessary (55, 59). Children and adolescents in Western countries are often exposed to substance use prevention intervention programs in school, which is strategic from a public health perspective, since all children attend school (60–64). In Sweden, providing educational information about illicit drugs is mandatory in primary and secondary schools, but the schools are free to elaborate on the teaching methods used (62–64). However, school-based prevention interventions have shown mixed results with regard to effects on cannabis use (8, 60); moreover, it remains to be clarified whether the potential effects of these programs last till adulthood (60). Evidently, school-based programs are not sufficient to prevent cannabis use in later life. Therefore, awareness campaigns and public information programs on the harmful effects of cannabis have been the cornerstones of substance use prevention among young adults who have left school; however, these have yielded questionable and sometimes adverse results (65–67). An excessive focus on the harmful consequences of drugs is perhaps one reason for the lack of desired effects (i.e., prevention of use) among the recipients of these programs (67). This might be particularly relevant in a Swedish context where the goal of the national policy on narcotics is “a society free from narcotics” (68). Information distributed from Swedish authorities and traditional print media generally portray cannabis as a potent and illegal drug contributing to social problems (69). Moreover, Swedish cannabis information symposia focus primarily on youth consumers, who are seen as particularly vulnerable to cannabis harmful consequences (69). Against this background, reasons for use, self-management or other harm reduction messages are not provided in Swedish public information on cannabis (39, 68). However, further research is needed to determine why public information on cannabis does not have the intended impact and how it should be modified to attain the desired effect, especially in the light of the international trends of deregulation and legalization of cannabis in recent years.

The decrease of legal restrictions, lowered risk awareness, and increasingly positive attitudes toward cannabis call for effective prevention measures besides, or instead of, prohibition, such as multicomponent environmental prevention programs that include effective information and communication on cannabis for the public (70). In order to carry out effective information-based prevention interventions, it is important to continuously monitor public attitudes toward cannabis use and prevention information.

The aim of the current study was to gain a deeper understanding of young adults' attitudes toward cannabis use and public prevention information. Specifically, the study aimed to answer the following questions: (1) How do young adults experience the risks associated with cannabis use? (2) Why do young adults use or abstain from using cannabis? (3) How do young adults view information about cannabis provided by society? (4) What kind of messages do young adults think can work to prevent cannabis use? (5) Which senders do young adults think would be effective in conveying a preventive message? (6) In what ways do young adults think information about cannabis should be provided?

To the best of our knowledge, this is the first study to examine attitudes toward cannabis use in parallel with attitudes toward public prevention information among young adults in Sweden. The results can guide the development of tailored information about cannabis for young people who cannot be reached by the universal prevention interventions in school settings in countries comparable to Sweden with regard to policy and cannabis use patterns.

## Materials and Methods

A qualitative study design encompassing individual in-depth interviews with young adults aged 18–29 years was employed to answer the research questions. Qualitative content analysis of the interview material was performed after verbatim transcription.

### Participants and Procedure

Purposeful sampling was used to recruit informants with varying backgrounds in terms of cannabis experience, sex, and socioeconomic background. The operationalization of cannabis experience was made by allocating informants who just tried cannabis once or a few times as well as people who were regular users in the category of “experienced” informants (tested), while those who had never tried cannabis was regarded as unexperienced (not tested). Sex was operationalized as man or woman, based on the informants' own definition. All informants chose one of these two categories when asked about their sex. The socioeconomic background was operationalized as residents in districts or municipalities with an average income of the adult population below or above the average of their respective municipality or district. Potential participants were contacted through social media (Facebook and Instagram), psychiatric clinics, the Police Authority, civil associations, and the researchers' own networks. People who were interested in participating were asked to contact the research team and were provided with information about what was required from them, that participation was anonymous, how the data were to be handled and presented, and that they could terminate their participation whenever they wanted. Informed consent was obtained and documented on tape (and later transcribed) before the interviews were conducted. The study was approved by the Swedish Ethics Review Authority (no. 2020-05669).

### Semi-structured Interviews

Two of the researchers (AS and PK) carried out the interviews using a digital video calling platform, using semi-structured interview guides adapted for four categories of informants: those aged 18–25 years with and without experience of cannabis use, and those aged 26–29 years with and without experience of cannabis use. The reason for grouping the participants into these age categories were the assumption that people older than 25 years of age have probably established their own lives and are less influenced by parents as well as friends than younger people, which is partly connected the fact that the development of the brain is not complete until approximately 25 years of age (71). Thus, the interview guides directed to younger and older informants, respectively, were slightly different. The interview questions were based in social learning theory and previous research on cannabis use, covering informants' own experiences of cannabis use, perceptions of risks with use, reasons for using or abstaining from use, and attitudes to publicly disseminated information about cannabis. Some examples of the interview questions are: “Would you like to say something about your own experiences of cannabis use?”, “What do you think are the reasons for using different types of cannabis?”, “Do you find any health risks associated with cannabis? If yes, what are they?”, and “Do you feel that you need information about cannabis? If so, what kind of information and from whom?”. The interviews, which took an average of 31 minutes to complete, were recorded with the approval of the informants and transcribed verbatim. After 32 informants had been interviewed, the interviewers judged that little or no new information would be obtained by interviewing additional people and the interview process was terminated due to perceived saturation (72). Background information about the participants is presented in Table 1. The final group of participants included 21 cannabis testers/users and 11 abstainers, 15 women and 17 men, 21 people aged 18–25 and 11 people aged 26–29, 18 people from municipalities or districts with an average income above the current average of the municipality or district, and 14 people from areas below the corresponding average.

**Table 1:** Background information about the informants.

		***N***	**%**
Age (years)	18–25	21	65.6
	26–29	11	34.4
Sex	Female	15	46.9
	Male	17	53.1
Socioeconomic status	Area income below average	14	43.8
	Area income above average	18	56.2
Experience of cannabis	Tested/user	22	68.8
	Not tested/non-user	10	31.2

### Content Analysis

Directed content analysis, inspired by Hsieh and Shannon (73) and Granheim and Lundman (74), was adopted to analyze the interview material. To increase the reliability of the analysis, a team-based approach was employed, involving three researchers of which two (AS and PK) worked closely together in the coding process, as described below (75). The NVivo 12 tool was used to structure the material and facilitate the analysis of the transcribed interviews. Initially, the two researchers who conducted the interviews (AS and PK) read through the transcripts repeatedly to find meaningful statements that could be grouped into preliminary categories and codes. An example of this grouping is presented in Table 2. The researchers were partly guided by the interview questions when looking for meaningful patterns, which according to Hsieh and Shannon (73) can be regarded as directed content analysis. With a directed approach, the analysis starts with a theory or relevant research findings as guidance for initial codes (73), which was the case in the current study. In the search for categories, information that could not be clearly linked to the interview questions was, however, also taken into account. During the coding process, the researchers discussed their findings and developed a preliminary coding scheme. Further review of the material and discussion with a third researcher (JG) generated additional revisions for categories and codes. Finally, an agreement was reached on the coding scheme presented in Table 3.

**Table 2:** An example of the content analysis.

**Meaning unit**	**Condensed meaning unit**	**Code**	**Category**
I think now when we're getting a little older, getting up to the age of 30, some of us, at least, are starting to have children and so, I think it [cannabis use] is something that is getting less and less common.	The propensity to use cannabis decreases with increasing age and as other interests and activities become important.	Competing activities or lack of interest.	Reasons to abstain.

**Table 3:** Final coding scheme.

**Categories**	**Risk awareness**	**Reasons to** **use**	**Reasons to abstain**	**Attitudes to public information**
Codes	Knowledge	Social milieu	Negative effects or consequences	Supply and availability
	Personal observations and experience	Individual factors or needs	Competing activities or lack of interest	Messages
		Positive effects	Family (including partner)	Senders Information transfer
			Prohibition	

## Results

The interview material generated four categories and 13 codes. In the following sections, the results of the interviews are presented by the categories identified in the content analysis: risk awareness, reasons to use, reasons to abstain, and attitudes to public information. Furthermore, subheadings corresponding to the codes generated during the analysis are used. Participants' direct quotes are presented in italics, followed by the sex and age of the corresponding informant.

### Risk Awareness

The experience of risks with cannabis use varied among the informants, but no clear difference was observed between users and non-users. Both groups appeared to base their perceptions of risks to a large extent on knowledge received from others, such as teachers in school, authorities, and *via* media. However, informants' risk awareness was also based on their observations and experiences with friends who used cannabis or their own experiences of using cannabis.

#### Knowledge

Most informants were aware that cannabis poses some form of health or social risks, but many lacked basic knowledge about the drug's negative effects. Some informants reported having previously received information about risks associated with drugs in school, for example, the effects of cannabis on the brain and body, while others did not remember any cannabis-related information from school at all.

*I actually have no direct memory of it [drug information in school]. We have certainly had that, but it is not something I remember. […] I have very poor knowledge of cannabis and drugs in general*.

-Female, 27 years

Informants who had tested cannabis had generally used the drug several times since their debut, and some of them did not perceive any significant risks associated with the drug, especially when using it “moderately.”

*I personally look at cannabis as alcohol. In the way that too much of it is absolutely not good, in the same way that too much of alcohol is not good*.

-Female, 23 years

#### Personal Observations and Experience

Informants who had not tested cannabis based their risk awareness mainly on public information in or outside school, the media, parents' views, and observations of friends; whereas, users, including those who had only tested it once and those who regularly used it, referred to a large extent to their own experiences when speaking about perceived risks. Some participants who remembered the information provided in school found it less credible because they perceived it as exaggerated and as depicting “horror scenarios.” Several participants leaned on information from acquaintances and media, and information gained from observing and communicating with friends who used cannabis, the latter leading to a higher as well as lower risk awareness.

*I have seen acquaintances who overuse, and you see how it [cannabis] affects those people negatively in different ways. For example, they become quite slow, and their reactivity does not improve immediately. It does not feel like it's a positive effect, at least not in those who overuse it*.

-Female, 28 years

Some informants mentioned that cannabis can lead to serious conditions, such as psychosis, and a few had themselves had unpleasant experiences related to cannabis use. One of the informants also described feelings of unreality and lasting symptoms several years after testing cannabis.

*However, these were symptoms of unreality, such as perceiving the world in other ways. […] I can still get that … It can come a little when… comes most under stress, when I am stressed about something*.

-Male, 18 years

The risk that cannabis use leads to the use of other drugs was also highlighted by several informants; one of them described his own case of drug abuse that began with his cannabis debut in his early teens. Another informant revealed that many of her friends had actually died as a result of drug abuse, which contributed to her refraining from cannabis and other drugs.

A majority of the informants had tested cannabis, and several of them continue to use it. All the informants knew at least one person who had tested or who uses the drug frequently, and many of them, especially cannabis users, perceived that cannabis use is common in the population, primarily based on their own observations.

*It [cannabis use] is common. It's very common. So the neighbor below us, they get high all the time*.

-Male, 19 years

Some informants concluded, through their own experiences or observations of other people, that it is possible to combine cannabis use with work productivity, school, and other daily activities, indicating a lower perception of risk, at least in the short term.

*I knew people who did it [used cannabis] regularly, every week, and at the same time did their studies very well. […] They got an A-grade on everything. […] So, they had A and felt great*.

-Male, 20 years

### Reasons to Use

The reasons for using cannabis described by the informants varied a lot, but were mostly related to the social milieu, individual factors or needs, and the positive effects of cannabis. Most of the reasons for using were reported by those who had tested or who used cannabis regularly.

#### Social Milieu

The social milieu seems to be important for cannabis use, especially when trying it for the first time. Several informants who had tested the drug mentioned that they had tried cannabis because it became available to them in some context.

*One of my friends had lived in Canada and had smoked cannabis when he was a teenager. And it was not something I had been interested in before. But I do not know, he was part of that gang of friends and then I think he got it in some way*.

-Male, 29 years

The overall impression was that young people feel that cannabis is easily accessible and they do not need to buy it themselves, because it can always be obtained through someone. Several informants reported that cannabis is used in different social contexts, and often in smaller groups. Cannabis does not appear to be a party drug that enhances a festive atmosphere; instead, it creates a relaxed atmosphere when hanging out with friends.

*Yes, it is nicer in smaller groups, smaller contexts, and safer environments, when there are fewer, that is when it's nicer*.

-Male, 29 years

As indicated above, some informants also considered cannabis harmless. This attitude appears to be based not only on their own experiences, but also on discussions with people in their immediate social environment.

*But I have thought about it a lot because I have not seen it as a heavy drug, or I… some people do not see cannabis as a drug at all, in my interactions. They see it as… almost like smoking a cigarette*.

-Male, 24 years

#### Individual Factors or Needs

Several informants reported individual reasons for using cannabis, such as unwinding, relaxing, and as an aid to sleep. Some described the drug as a way to find peace, achieve a good feeling, or escape reality.

*Then, it's just like this, escape reality a little, and feel relaxed. And do not think so much about what is happening. An escape, sort of*.

-Female, 23 years

Another aspect of cannabis use was rooted in mental health issues, where it was used as a way of managing or reducing anxiety.

*Yes, I can well imagine that it's… can have a calming effect and be nice. […] reduce anxiety a bit*.

-Female, 27 years

Another reason for using, especially for testing cannabis, was curiosity. As mentioned above, the drug use debut is not usually planned in advance, but is done in the moment when the opportunity arises.

*When we were in Copenhagen, it was a fun thing. And man… I am so curious so I think it's fun to test, but I know it's really stupid too*.

-Male, 24 years

#### Positive Effects

Some informants revealed that cannabis contributes positively to a calm and cozy atmosphere in social settings.

*I have only positive experiences. I think it's much… It's much nicer and like… yes, but calmer and cozier to hang out when you have used cannabis ‘rather than' alcohol, for example. It becomes much more of a… to sit at home and laugh and talk and maybe watch a movie or something like that*.

-Female, 22 years

Positive effects are also experienced when users are on their own. One of the informants expressed two types of positive emotions in the following way:

*I become happy and feel smart and wise and strong and want to invent something, do something fun, and take a trip. Maybe go for a walk, paint, or something. [and further] I get more tired and drowsy and so, but not in a negative way, but I get a little sleepy, a little cuddly, a little cozy. I might want to watch a movie or create something in a quiet environment. So, I get two different effects*.

-Female, 19 years

### Reasons to Abstain

The informants' key reasons for abstaining from cannabis use were the risks of health and social problems, such as mental illness, negative neuropsychological consequences, losing control, and a connection to (or promotion of) organized crime. The user's family or partner can also be an important factor in the decision to abstain, along with the (Swedish) legislation and penalties associated with cannabis use.

#### Negative Effects and Consequences

Many of the informants had heard of or observed the negative effects or consequences of cannabis, including those who had tested the drug. In some cases, negative experiences during the first time or later had led to the choice of giving up the drug, or using it less often.

*I am probably one of the few people who have experienced a traumatic scenario from the first time. […] The only thing I can do is to keep my distance from it and so. I have no problems with that now. I'm terrified of it!*

-Male, 18 years

Furthermore, a couple of the informants pointed out that users' lack of ability to finance their drug use may lead to fights and physical aggression, or being forced to start selling the drug themselves.

*The problem here is that you can very easily become… get into it, and get stuck. So if you don't have these financial opportunities, if you are not financially able to pay and you get stuck, then it can lead to huge catastrophic “penalties.” So you can become a criminal, you can become a part of it [the dealing] and sell and buy*.

-Male, 20 years

#### Competing Activities or Lack of Interest

Some informants expressed that they had no interest in using cannabis. This applied both to those who had tested it and those who had never used cannabis.

*I have no interest in consuming cannabis, so I feel rather that it's something I want to distance myself from*.

-Female, 27 years

The absence or lack of the expected effects during their cannabis debut was also presented as a reason to abstain from use.

*I actually barely felt anything. It was well that you laughed a little more maybe, but I did not feel anything, I thought it was completely useless*.

-Male, 24 years

Moreover, the responsibilities of raising children, work, and other competing interests and activities also contributed to cannabis use becoming less appealing with increasing age. In addition, the feeling that it is cool to use cannabis decreased as one approached the age of 30.

*I think now that we are getting a little older, getting up to the age of 30, some of us are starting to have children and so on, so it's I think it [cannabis use] is something that's getting less common*.

-Male, 29 years

#### Family or Partner

Family norms did not appear to be central to the choice of trying cannabis. In some cases, however, parents and the home environment seemed to influence abstinence from the drug, as expressed by one of the abstainers:

*The whole family probably thinks it is a little tangled, sort of, if you may say so. I still understand somehow that you want to escape your problems quickly, but it feels… it's not the right way. It feels like those who take drugs may not think about the consequences. I think there are many consequences. We have not talked much about drugs at home, but it has always been a “big no no” to use drugs*.

-Female, 23 years

In the case of ongoing use, a partner's demand for abstinence was also mentioned as a reason to stop using cannabis.

*The reason to stop using cannabis would probably be that the girlfriend wants us [the informant and his girlfriend] to stop*.

-Male, 24 years

#### Prohibition

The Swedish legislation prohibiting the use and handling of cannabis was reported by most informants as a prominent reason to abstain from use, and that the fact that cannabis is illegal served as a signal that it is unhealthy and dangerous to use.

*In addition to the negative consequences of any stigma and sanctions associated with the ban, the ban also seems to serve as a signal that cannabis is “dangerous.” It's easy to get caught and then you become addicted and then it's very difficult to stop […] and then you can imagine that why is it [cannabis] illegal? This is probably because it is easy to get caught up in*.

-Male, 21 years

### Attitudes to Information

The informants provided their views on several aspects of public information about illicit drugs in general, and cannabis in particular, including supply and availability, messages, senders, and the way the messages are transferred to the target group.

#### Supply and Availability

Overall, the informants felt that they had not received any information about cannabis from competent authorities in the society. Only a few of them remembered the information disseminated in school, and those who had received information did not perceive it to be sufficient.

*So I kind of do not think you get that much information, to be honest. It's mostly… So the school does not address anything at all, I think*.

-Female, 23 years

Several informants also mentioned that information was publicly available online, for example on various authorities' websites, but young people had to search for it themselves.

*If you go to an authority's website, it says [provides information on cannabis]. However, not everyone may make an active choice to go in and check*.

-Female, 23 years

Sometimes, the problem was not a lack of information, but the fact that it was difficult for adults to reach out to young people, because the youth tend to ignore risks.

*I do not know, I think it's difficult… or I think it's a difficult thing over all, to reach out to young people, because they think more that they are immortal and that one should not listen to adults*.

-Male, 24 years

At the same time, some informants stated that there is definitely a need for more information.

*Yes, it would be good… it would be good. I think there are many who do not know, or as I said, I am not a hundred percent sure about what cannabis does to you. I am not so familiar with… I have never been interested in drugs, but it… I think it's good to know about drugs*.

-Male, 24 years

A few of them also mentioned that there is a taboo around conversations about cannabis and drugs in general, which may increase the risk of harm among young people.

*Yes, but that it is still a bit taboo. And that we do not talk about it as much as we would need. […] And I think there are many more who just see it like this: “Oh, that's a fun thing.” So, I think very few people understand the meaning of it and the risks. Thus, the information I think can be improved*.

-Female, 23 years

#### Messages

Some of the informants revealed a distrust toward the adult world and authorities, implying that adults emphasize the negative aspects of cannabis use and try to scare young people to prevent them from using. Such an approach can perhaps work for children and people in their early teens, according to one of the informants, but when children grow older and have friends who have tested the drug or test it themselves, they question the credibility of the information they had received. Several participants requested information in the form of valid facts and clarity about the specific effects of cannabis use, for example, the changes that take place in the body and brain when using cannabis.

*Yes, I think people need information and accurate information about it. I think that this is important. It is not moralizing, and it is very clear what the negative effects are. And that's quite… yes, objective… with as good research data as possible, to show the negative effects that it can have*.

-Male, 29 years

An informant also mentioned that the Swedish society's zero tolerance policy for illicit drugs creates problems in communication and a lack of transparency in information dissemination.

*I think it is very difficult for authorities to seem credible in external communication if you have a form of zero tolerance for drugs. […] I am not sure it's a problem that lies in communication, but I think it's a problem that lies in the legislation, if I am honest*.

-Male, 29 years

Some informants also expressed a need for information on how to use cannabis safely, for example, how much of it can be consumed in large quantities and descriptions of what can happen on consumption.

*You still want to know before you start using… When you test it, it's always good to know what you are going into*.

-Female, 19 years

Some informants believed that many young people receive information and form their impressions of cannabis through music, film, television, and social media, which can lead to an incorrect or glamorized view of the drug. Artists using cannabis may be perceived as “cool” and a normalization of cannabis use may occur.

*It feels like children learn more about drugs from movies. […] All these guys who rap today and have face tattoos in videos with drugs and stuff. […] I think young people get a completely wrong idea of drugs*.

-Male, 24 years

#### Senders

The informants emphasized that the sender of cannabis-related information is important for the information to be perceived as credible. They indicated that they preferred someone who “knows what he or she is talking about” and someone they could look up to.

*The sender I think is A and O. […] Someone who has experience [of cannabis use], but perhaps together with a person in a lecture who is known for something, who people look up to*.

-Female, 23 years

Several of the informants reiterated the fact that the best person to speak about cannabis is someone with personal experience of using, and preferably someone with whom the target group can identify, for example, a person of the same age.

*I think they listen more if there is someone who is maybe 30, 35 and is a former drug addict but has gotten out of it, and that they tell about their experiences*.

-Male, 24 years

One of the informants stated that everyone has the right to the same and equal information regardless of where they live and where they come from, pointing to schools as a key sender of information related to drugs. The informants also addressed the role of parents as important in providing information about drugs, although parents are not “public senders.”

*Yes, it's really the parents' responsibility. However, I think that not all parents may do so. Because I am not… I am not ethnically Swedish, and my parents may not even fully know what cannabis is specifically. So, I still think that the school should be able to take some responsibility for that really. Thus, all children from different ethnicities could learn*.

-Female, 23 years

The informants also pointed out that there are advantages in employing a neutral sender with competence, that is, deep knowledge about the subject, rather than, for example, a teacher who normally teaches other subjects.

*What I think is that it would have been best to bring in a neutral sender who then informs about it [cannabis]. So that it does not happen that, “Gunilla, who teaches English and Swedish, should start talking about cannabis.” If so, I think it will be like this: “Yes, but what does she know?”*

-Female, 23 years

#### Information Transfer

An idea put forward by some of the informants was that the involvement of famous people, such as “influencers,” could be a successful alternative for reaching out with information to young people. Authorities could, for example, establish collaborations with celebrities or other people who already function as role models.

*There are many influential role models. So really, maybe you can train the influencers so that they can provide good information*.

-Female, 23 years

The importance of having an open dialogue when delivering information was also underlined, particularly by making young people feel that their experiences are being taken into account.

*Try to enlighten more and encourage discussion among students. And maybe… Yes, and based on them and their experiences like this: “Yes, but what do you think? What do you feel then?” and have it more open*.

-Female, 23 years

The informants regarded public information as a more or less realistic way to prevent people from testing cannabis, but expressed skepticism about the possibility of making regular users quit by informing them about the harmful effects.

*No. I do not think so. I think you have to decide for yourself to be able to quit. It is not up to anyone else. I can only think of myself because I smoke. If someone else were to say to me “quit smoking cigarettes.” It's not like I would quit because someone says so… I know what health risks there are with smoking, and yet I continue. So, it has to come from myself if I want to quit. I think it's the same way with cannabis too*.

-Female, 23 years

## Discussion

This study aimed to gain a deeper understanding of young adults' attitudes toward cannabis use and public prevention information about cannabis.

### Summary of Results

Both cannabis users and abstainers perceived some risks with cannabis; however, for many users, the positive effects seemed to outweigh any expected harm, although both users and non-users described both positive and negative effects. Furthermore, public information was perceived as less credible by the participants because of an excessive focus on harmful effects, which they considered to be connected to the zero tolerance policy for cannabis use in Sweden. The informants expressed a desire for neutral facts about the effects of cannabis, delivered by someone with deep knowledge of the subject. Moreover, they felt that, to improve credibility, prevention information should be delivered by a sender they can look up to, or a person with whom young people can identify. The informants also stated the importance of dialogue with the target group and of taking young people's experiences into account when providing information about cannabis.

### Comparison With Previous Research

The informants in this study were well aware of the risks associated with cannabis use, although several of them lacked detailed knowledge of cannabis-related effects and a few regarded cannabis as being almost harmless. To the extent that risks were recognized, they did not prevent a large number of the participants from testing cannabis or from using it regularly. Possibly the information that they received did not sufficiently emphasize the risks associated with the drug, or the target group did not care about the risks, the latter perhaps more likely since the public information on cannabis in Sweden to a large extent focus on risks (69). As mentioned by a participant, young people may believe they are “immortal” and, thereby, are almost immune to risk information. Nevertheless, cannabis use in Sweden is still less common than in many other countries (32), which may be linked to the risk of penalty on violating the Swedish ban on using or handling cannabis (28), or the perception that the ban signals dangers connected to cannabis. In the current study, about 70% of the informants reported having personal experience of cannabis use, and several of them believed that the drug was commonly used in society. A national health survey conducted by the Swedish Public Health Agency in 2018 showed that 17% of women and 25% of men aged 16–29 years had tried cannabis at least once (34), a significantly lower figure than the proportion in this study. The large number of informants with cannabis experience in the current sample could be attributed to the fact that people who have tried cannabis may be more interested in participating in a study on cannabis than people who do not have this experience. It was also a conscious strategy to include users as well as non-users in the study, in order to learn the different reasons for using and explore how users perceived prevention information, since users are more likely to influence peers who have not yet tested the drug (44–49).

It was observed that any awareness of risks, including the risk of penalty when violating the ban, did not prevent a significant proportion of young adults from testing and continuing to use cannabis. In a departure from the rational choice theory (51, 52), the perceived harm did not appear to outweigh the expected utility of using cannabis. The informants highlighted several motives for using cannabis, such as enhancing socialization with friends, increasing personal well-being, and counteracting negative feelings and mental health problems, which are reasons previously reported by young users (35, 36). The social aspect of substance use was recently highlighted in a study on Swedish students aged 15–19 years (57) in a slightly different way; among these younger individuals, peer pressure appeared to be a more prominent social motive than promoting positive experiences when hanging out with friends, whereas, the latter was emphasized more in the current study. The availability of cannabis, however, seems to be important for both adolescents and young adults when using the drug for the first time, as shown in the current study as well as the study on Swedish students (57). Since cannabis use is prohibited by the law in Sweden, the social context with peers who can provide the illegal substance, explain how to use it, and relate the expected effects may be even more important for the initiation of cannabis use among Swedish youth compared to young people in countries where cannabis can be purchased legally and information about its use and effects can be obtained easily.

Given that young adults have access to cannabis, have several reasons to use it, and perceive a number of positive effects associated with it, it is a significant challenge to prevent them from using it by providing information about its risks and harmful effects. Research suggests that presenting information alone has limited effects on behavior, and that a comprehensive perspective is more effective for substance use prevention, such as multicomponent programs where information is one of several components (65, 76). Such interventions address supply and demand, the latter being influenced by societal norms and values (70). In line with this, participants in the current study were skeptical of the fact that established cannabis users could be persuaded to stop using the drug by only informing them about the associated risks. Nevertheless, several of them actually wanted more information about cannabis, preferably, details about the effects of cannabis on the body and brain. This opinion was expressed by those who had never tried cannabis as well as those who regularly used it, the latter partly because they wanted to use the drug “safely.” Previous studies support the health benefits of self-management and protective strategies when using cannabis (39, 77–79). Whether such benefits could outweigh the results of a strict focus on risks with regard to public health in a country like Sweden, where the prevalence of cannabis still is fairly low, remains to be explored, and also which implications for information interventions such knowledge might generate. Based on our results, future studies, in any case, need to examine the possibility that preventive messages need to be formulated and directed at specific target groups rather than as a general message to the entire population.

Although the current study managed to recruit cannabis users who gladly shared their experiences (admittedly anonymously), some informants stated that the present “taboo” around cannabis in Sweden, partly generated by the prohibition by law, counteracted a nuanced communication from the authorities, thereby impeding the credibility of public information. This view is in line with previous research, indicating that an overly one-sided focus on the harmful consequences of cannabis may impede the intended effect of the preventive message (57, 67). In congruence with the desire for nuanced information on cannabis, participants in the current study asked for a dialogue with the information provider or sender, in which their own experiences were taken into account. This aspect has previously been emphasized by Moffat et al. (80), suggesting consulting youth themselves for reality-based content in information-based cannabis prevention interventions. Additionally, research on selective prevention interventions, such as motivational interviewing to promote behavioral change in risk groups, highlighted the importance of a non-judgmental and less moralizing approach which stayed neutral to the actual behavior, including an assessment of advantages and disadvantages (81, 82). This approach may also apply to universal information prevention interventions, as supported by the current study as well as by previous research (80). Finally, the participants also highlighted the importance of a credible information provider or sender. Credibility can be gained either through having deep knowledge of the subject, an appearance of authority, or personal experience of the drug. These opinions were also shared in the interview study on Swedish students (57) with slightly younger informants. It may be debated whether people with a personal experience of substance use can be more objectively credible or helpful, but young people seem to place their trust in them. However, it is important to note that the students in the previous study (57) emphasized that the person with personal experience of using drugs should not be just anybody, for example, “a heroin addict who talked about having found Jesus,” but someone with whom they could identify. Credibility can also be increased through identification with the sender by similarity in age. In the current study, it was apparent that informants looked for reliable information from friends and acquaintances. Apart from similarities in age and life situation, friends may provide credibility through their established interpersonal relationships. Finally, the informants pointed out that famous people, such as “influencers,” can be credible senders and that public authorities should cooperate with them when communicating information about cannabis. It is unlikely that this proposal is based on influencers' potential knowledge of cannabis, but on the fact that young people identify with them because they represent the values that the youth hold, and that they manage to establish some kind of friendship with their followers, creating a feeling of benevolence that may increase credibility (83).

### Strengths and Limitations

To our knowledge, the present study is unique in that it combines interviews with Swedish young adult users and non-users, exploring their attitudes toward both cannabis use and information to prevent use. This study has several additional strengths that should be noted. First, it was based on interviews with informants representing a wide range in terms of age, sex, socioeconomic background, and experience of cannabis use, thus generating rich material highlighting many aspects of young people's attitudes toward cannabis. Second, the research team had extensive experience of research on drug-related issues in society, including cannabis, both in terms of health and policy aspects. Two of the researchers conducted the interviews themselves, which enabled professional interviews with relevant follow-up questions as well as a deep knowledge of the material even before the qualitative content analysis began. Furthermore, a team-based analysis process was used to ensure reliability of the results. However, this study also has some limitations. The recruitment process was voluntary, which entailed a risk of distorted selection because people who wanted to participate may differ in important ways from those who did not want to participate. In the current study, there was an overrepresentation of cannabis users in relation to non-users; this was somewhat surprising in light of the strict Swedish law against cannabis, which was assumed to deter users from participating. Another limitation is the quite sparse background information about the sample, limiting the possibility to obtain a more comprehensive profile of the informants. Finally, in an interview situation, there is a risk that the interviewee may respond in a way that they believe the researcher expects them to (42).

## Conclusion

Current risk awareness associated with cannabis use among young adults is insufficient to prevent actual cannabis use. Therefore, multicomponent drug prevention programs, where information is one of the components or strategies, should be implemented, combining a firm and fact-based focus on risks with recognition of cannabis' short-term desired effects, delivered by credible senders with authority or those with whom young adults can identify.

## Data Availability Statement

The data are available from the Centre for Psychiatry Research, a collaboration between the Karolinska Institutet and Region Stockholm, but restrictions apply to their availability, as they were used under ethical permission for the current study, and therefore, are not publicly available. However, data are available from the authors upon reasonable request and with permission from the Centre for Psychiatry Research.

## Ethics Statement

The research protocol was approved by the Swedish Ethics Review Authority and was conducted according to the principles expressed in the Declaration of Helsinki and all participants provided written informed consent.

## Author Contributions

PK: conceptualization, methodology, investigation, data curation, formal analysis, project administration, writing–original draft, and writing–review and editing. AKS: conceptualization, methodology, investigation, data curation, formal analysis, project administration, and writing–review and editing. JG: conceptualization, methodology, formal analysis, supervision, funding acquisition, and writing–review and editing. All authors contributed to the article and approved the submitted version.

## Funding

This work was funded by the Stockholm Health Care Administration. The funding body had no role in the study design, data collection, analysis, data interpretation, or writing of the manuscript.

## Conflict of Interest

The authors declare that the research was conducted in the absence of any commercial or financial relationships that could be construed as a potential conflict of interest.

## Publisher's Note

All claims expressed in this article are solely those of the authors and do not necessarily represent those of their affiliated organizations, or those of the publisher, the editors and the reviewers. Any product that may be evaluated in this article, or claim that may be made by its manufacturer, is not guaranteed or endorsed by the publisher.
